# The Effects of Antiepileptic Medications on Lipid Profile, Thyroid Panel, and Vitamin Level

**DOI:** 10.7759/cureus.11005

**Published:** 2020-10-17

**Authors:** Hüseyin Büyükgöl, Muzaffer Güneş

**Affiliations:** 1 Neurology, Konya Ticaret Odası Karatay University, Konya, TUR; 2 Neurology, Aksaray University Training and Research Hospital, Aksaray, TUR

**Keywords:** antiepileptic, lipid, thyroid, vitamins

## Abstract

Background: Conventional antiepileptic drugs (AEDs) have been used for many years to treat epilepsy, and physicians are generally familiar with their side-effect profiles and potential drug interactions. However, AEDs affect patient vitamin and mineral levels in a manner that is not well understood. The goal of this study was to determine the relationship between AEDs and patient vitamins and mineral levels.

Materials and methods: We conducted a retrospective analysis of liver enzyme levels, thyroid hormone levels, lipid profiles, and vitamin values (e.g., B12 and folic acid) in patients treated with carbamazepine, valproic acid, or levetiracetam at our institution. Patients were included in the study if their medical data included total cholesterol, triglyceride levels, low-density lipoprotein (LDL), and high-density lipoprotein (HDL) levels obtained on follow-up at least three months after the start of antiepileptic treatment with carbamazepine, valproic acid, or levetiracetam. Patients were grouped according to the antiepileptic drug used. We analyzed liver thyroid function tests, lipid profiles, levels of B12, and folic acid levels using laboratory test results and compared the findings from each group.

Results: Carbamazepine, valproic acid, and levetiracetam did not change the levels of liver enzymes such as aspartate transaminase and alanine aminotransferase (p values respectively: .802, .094). Cholesterol and LDL levels were lower in patients using carbamazepine (p values respectively: .005, .005), and no significant difference was observed for HDL and triglyceride levels (p values respectively: .400, .091). While thyroid-stimulating hormone levels were significantly higher in patients on medication than the control group (p=.007), the levels were still within reference ranges. No significant difference was found between tri-iodothyronine and thyroxine levels (p values respectively: .065, .053). The levels of B12 and folic acid were observed to be high in the group using carbamazepine (p values respectively: .049, .004).

Conclusion: Valproic acid and carbamazepine do not induce a significant increase in liver enzymes compared to levetiracetam, a new-generation antiepileptic medication, and they had no impact on lipoproteins such as HDL that are protective against coronary artery disease. These medications do not affect the levels of thyroid hormones in comparison to levetiracetam and the control group. Although carbamazepine and valproic acid are metabolized in the liver, liver enzyme monitoring is required; they have only affected liver enzyme values as much as levetiracetam and the control group.

## Introduction

Conventional antiepileptic drugs (AEDs) have been used to treat patients with epilepsy for many years. These drugs are well-defined, and their side-effect profiles and potential drug interactions are well understood by the medical community. Thirty years ago, a newer generation of AEDs became available that were as effective as the conventional antiepileptic agents but with fewer adverse effects [1]. Many studies have investigated the effect of AEDs on serum lipid levels [2]. AEDs can cause hyperlipidemia by inducing the p450 enzyme system in the liver, which may predispose a patient to atherosclerosis [3]. Long-term use of AEDs can result in a variety of adverse metabolic and endocrine effects [4]. AEDs also influence the levels of some vitamins and minerals. Long-term use of AEDs increases the risk of folate deficiency by lowering folate levels in approximately half of epilepsy patients [5]. Therefore, the goal of this study was to evaluate the effects of carbamazepine, valproic acid, and levetiracetam on liver enzyme levels, thyroid-stimulating hormone (TSH) levels, lipid profiles, and vitamin values (e.g., B12 and folic acid) in the epilepsy patients treated in our hospital.

## Materials and methods

We conducted a retrospective review of the medical records of patients treated from May 2014 to December 2016 in the Neurology outpatient clinic of Aksaray University Medical School Training and Research Hospital. Our institutional review board approved the study. AED use among patients with the diagnosis of epilepsy were included in the study. The study included patients whose medical data included measurements of total cholesterol (TC), triglyceride (TG), low-density lipoprotein (LDL), and high-density lipoprotein (HDL) obtained on follow-up at least three months after the start of either carbamazepine, valproic acid, or levetiracetam. We excluded patients with a history of diabetes, hypothyroidism, hyperlipidemia, stroke, and coronary artery disease.

We evaluated liver and thyroid function tests, lipid profiles, and vitamin B12 and folic acid levels according to patient laboratory records. Each group was evaluated based on the AED used.

We used IBM Statistical Package for Social Sciences (SPSS) Statistics version 20 (IBM Corp., Armonk, NY, USA) for statistical analysis. Chi-square test was used for intergroup comparison in terms of sex. The Kruskal Wallis and Mann Whitney-U tests were used to compare data between the groups. Statistical significance was accepted as p<0.05.

## Results

A total of 124 patients were included in the study. Twenty-five patients were treated with carbamazepine monotherapy, 25 patients were treated with valproic acid monotherapy, and 25 patients were treated with levetiracetam monotherapy. Our control group included 49 healthy volunteers of similar age and sex ratio to the test groups. The average age of the participants was 28.8 years.

No significant difference was observed between groups with regards to age (p=0.553) or sex (p=0.935). Carbamazepine, valproic acid, and levetiracetam did not significantly affect liver enzymes aspartate transaminase (p=0.802) and alanine aminotransferase levels (p=0.094).

Cholesterol (p=0.005) and LDL (p=0.005) values were significantly lower in carbamazepine users than in the other groups. Although a significant difference was observed in the levels of LDL (p=0.05) and TC (p=0.05) in the cholesterol panel (low in carbamazepine group), there was no difference in terms of the levels of TG (p=0.400) and HDL (p=0.091). In thyroid hormone levels, a significant difference was found in the levels of TSH (p=0.007) for all three medications studied (but still within the reference range), while there was not a significant difference in tri-iodothyronine (FT3; p=0.053) and thyroxine (FT4) levels (p=0.065).

Patients in the carbamazepine group had significantly higher B12 (p=0.049) and folic acid vitamin levels (p=0.004; Table 1) compared to the other groups.

**Table 1:** The effects of antiepileptic medications on lipid profile, thyroid panel, and vitamin level ALT: alanine aminotransferase, AST: aspartate aminotransferase, GGT: gamma-glutamyl transferase, LDL: low-density lipoprotein, HDL: high-density lipoprotein, TSH: thyroid stimulating hormone, FT3: tri-iodothyronine, FT4: thyroxine

Analyte	Control group	Carbamazepine	Valproic acid	Levetiracetam	P
AST	20.08±9.46	16.96±5.55	17.48±3.00	17.92±6.18	.802
ALT	19.6±8.80	15.40±8.69	18.24±6.41	19.88±14.23	.094
GGT	19.06±6.36	24.52±19.23	29.84±19.91	48.72±66.05	.022
Cholesterol	190.98±30.44	165.92±32.96	179.76±29.15	194.72±45.45	.005
LDL	111.27±30.44	85.96±36.00	94.92±29.48	108.60±41.50	.005
HDL	53.81±15.27	54.48±18.02	59.28±15.44	56.68±23.83	.400
Triglyceride	109.14±39.08	128.16±75.75	113.08±70.30	142.56±74.13	.091
TSH	1.62±0.91	2.16±1.22	2.21±1.02	2.44±1.19	.007
FT3	1.08±0.25	1.1792±0.24	1.01±0.21	1.16±.43	.065
FT4	2.78±0.66	3.09±0.46	2.85±0.36896	2.80±0.55	.053
B12	292.47±112.84	379.92±150.16	281.44±67.16	299.12±112.34	.049
Folic acid	6.49429±2,26	8.45±2.49	6.87600±2.500005	7.86±2.54	.004

## Discussion

Epilepsy is a disease requiring long-term, sometimes lifelong, treatment [6]. Although epilepsy medication plays an important role in prognosis, effective classification of epileptic seizures and syndromes provides the best knowledge of prognosis. In a study by Sarmast et al., the authors recommended the use of a multidimensional classification model that incorporates the clinical semiology, disease location, aetiology, and associated comorbidities. The benefit of this model results in better patient outcomes [7]. Long-term antiepileptic therapy affects the frequency and development of cardiovascular diseases [8]. Increases in the levels of serum cholesterol, LDL, and TG are risk factors in the development of coronary artery disease and atherosclerosis, while HDL levels have a protective role against these diseases [9,10]. The comprehensive evaluation of lipid values allows for assessing the potential adverse effect profiles of AEDs and, specifically, their relationship with ischemic heart disease. The studies from the period when carbamazepine was not commonly used reported that the incidence of ischemic heart disease is significantly increased in patients with the diagnosis of epilepsy [11]. Various results have been observed in studies regarding lipid panel. HDL cholesterol level was significantly higher in patients on carbamazepine than the control group in a study done by Apak et al. [12]. In our study, although cholesterol and LDL levels were significantly low, no significant difference was observed in the levels of HDL and TG. Although the TSH levels were increased in patients on each medication, TSH levels remained within the reference range. FT3 and FT4 levels showed no significant difference compared to the control group. In line with this, Bozdagan et al. reported that the frequency of subclinical hypothyroidism in patients who are on carbamazepine is equal to that of the healthy population, and there is no need for monitorization with thyrotropin-releasing hormone stimulation test with regards to subclinical hypothyroidism; follow-up with thyroid function tests is sufficient [13].

The effect of AEDs on folic acid levels is variable. Folic acid deficiency is seen in patients on AEDs that particularly cause enzyme inductions [14-17]. In our study, inconsistent with the literature, levels of B12 and folic acid were higher in patients taking carbamazepine. This may be because these vitamins are prescribed to the patients using carbamazepine in internal medicine clinics.

A retrospective design and small population limited our study. Because we were unable to determine many parameters before the initiation of the treatment, we were not able to detect the confounding factors or analyte levels over time. However, the selection of the patients who regularly come to the outpatient clinic and have been using their medication for at least three months and evaluating data from patients that are only on monotherapy are strengths of the study.

## Conclusions

While valproic acid and carbamazepine, which are old-generation AEDs, did not cause a significant increase in liver enzymes when compared to levetiracetam, a new-generation AED, they did not have an effect on HDL-like lipoproteins that are protective against coronary artery disease. These drugs do not influence the levels of thyroid hormones when compared to levetiracetam and the control group. Although carbamazepine and valproic acid are metabolized in the liver, physicians should still continue to monitor patient liver enzyme levels; these medications have only affected liver enzyme values as much as levetiracetam and control group.
